# Synthesis and structure-activity relationships of cerebroside analogues as substrates of cerebroside sulphotransferase and discovery of a competitive inhibitor

**DOI:** 10.1080/14756366.2020.1791841

**Published:** 2020-07-13

**Authors:** Wenjin Li, Joren Guillaume, Younis Baqi, Isabell Wachsmann, Volkmar Gieselmann, Serge Van Calenbergh, Christa E. Müller

**Affiliations:** a Department of Pharmaceutical & Medicinal Chemistry, PharmaCenter Bonn, Pharmaceutical Institute, University of Bonn, Bonn, Germany; b Laboratory for Medicinal Chemistry, Gent, Belgium; c Department of Chemistry, College of Science, Sultan Qaboos University, Muscat, Oman; d Institut für Biochemie und Molekularbiologie, University of Bonn, Bonn, Germany

**Keywords:** Capillary electrophoresis (CE), galactosylceramide sulphotransferase (CST), enzyme assay, metachromatic leukodystrophy (MLD), competitative inhibitor, galactosyl ceramide analogues

## Abstract

Metachromatic leukodystrophy (MLD) is a rare genetic disease characterised by a dysfunction of the enzyme arylsulphatase A leading to the lysosomal accumulation of cerebroside sulphate (sulphatide) causing subsequent demyelination in patients. The enzyme galactosylceramide (cerebroside) sulphotransferase (CST) catalyses the transfer of a sulphate group from 3′-phosphoadenosine-5'-phosphosulphate (PAPS) to cerebrosides producing sulphatides. Substrate reduction therapy for arylsulphatase A by inhibition of CST was proposed as a promising therapeutic approach. To identify competitive CST inhibitors, we synthesised and investigated analogues of the substrate galactosylceramide with variations at the anomeric position, the acyl substituent and the carbohydrate moiety, and investigated their structure–activity relationships. While most of the compounds behaved as substrates, α-galactosylceramide **16** was identified as the first competitive CST inhibitor. Compound **16** can serve as a new lead structure for the development of drugs for the treatment of this devastating disease, MLD, for which small molecule therapeutics are currently not available.

## 1. Introduction

**Figure 1.**
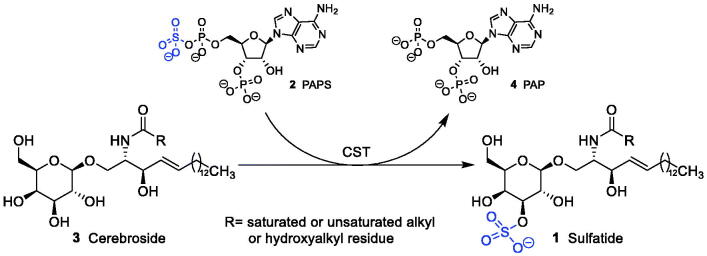
Sulphatide synthesis by CST: galactosylceramide (cerebroside) is converted to sulphatide by CST in the presence of PAPS as sulphate donor.

Metachromatic leukodystrophy (MLD) is a rare genetic disease characterised by a dysfunction of the enzyme arylsulphatase A^1^. This defect leads to the lysosomal accumulation of cerebroside sulphate (sulphatide, **1**) in various cells such as tubular kidney cells, bile duct epithelia, some neurons, oligodendrocytes and Schwann cells. In particular accumulation in the latter two results in progressive demyelination finally causing lethal symptoms in patients. Recently haematopoetic stem cell-based gene therapy has been shown to be effective in patients in early preclinical states of disease only^2^. Thus, there is an urgent need to develop alternative strategies to treat MLD. One of these strategies is substrate reduction therapy in which galactosylceramide (cerebroside) sulphotransferase (CST; EC 2.8.2.11), the enzyme which synthesises sulphatide, is inhibited. This would diminish the load of accumulated sulphatide in the patient. Such a strategy has been shown to be effective in Gaucher disease, another lysosomal sphingolipid storage disorder^3^. Inhibition of galactosylceramide sulphotransferase has been proposed as a promising new therapeutic strategy for the treatment of MLD^1^^,^^4^. CST catalyses the transfer of a sulphate group from the coenzyme 3′-phosphoadenosine-5′-phosphosulphate (PAPS, **2**) to galactosylceramide (**3**) yielding galactosylceramide sulphate (**2**) and adenosine-3′,5′-bisphosphate (PAP, **4**) (Figure 1)^5–7^.

**Figure 2.**
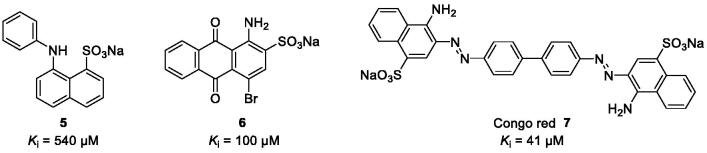
Chemical structures of aromatic dyes known as CST inhibitors competing with the co-substrate PAPS.

We, therefore, aim at developing CST inhibitors to reduce the biosynthesis of sulphatide to prevent sulphatide aggregation in the central and peripheral nervous system^7^. Sulphation is a widely observed biological reaction conserved from bacterium to human that plays a key role in various biological processes^8^^,^^9^. Deficiencies due to the lack of the ubiquitous sulphate donor PAPS are lethal in humans^8–10^. A large group of enzymes called sulphotransferases catalyses the transfer reaction of the sulphuryl group of PAPS to the acceptor group of numerous biochemical and xenobiotic substrates^11^. Structure-based sequence alignments based on X-ray crystal structures indicate that the PAPS-binding site is conserved^8^^,^^9^^,^^12^. Therefore, competitive inhibitors for the CST substrate galactosylceramide are expected to have less side effects compared to inhibitors competing with the co-substrate PAPS. However, so far only few weakly potent, non-selective CST inhibitors have been described (Figure 2)^13^.

A reaction mechanism between the enzyme, the co-substrate PAPS, and the substrate galactosylceramide has been proposed based on crystal structures^14–16^. A transition state mimetic might efficiently inhibit the CST-catalysed reaction^17^. In the present study, we synthesised and investigated a series of substrate analogues with the aim to study their structure–activity relationships (SARs) as substrates and to identify competitive inhibitors.

## 2. Materials and methods

### 2.1. Commercial compounds

Psychosine was purchased from Sigma (Steinheim, Germany). Galactosylceramide and glucosylceramide were obtained from Matreya LLC (Pleasant Gap, PA); according to the supplier, cerebroside consists of a mixture of saturated or unsaturated fatty acid residues (C16:0, C18:0, C20:0, C22:0, C23:0, C24:0–C27:0, C24:1–C27:1) or hydroxyacyl residues (C18:0(2–OH), C20:0(2–OH), C22:0(2–OH), C23:0(2–OH), C24:0(2–OH), C24:1(2–OH), C25:0(2–OH)) and glucosylceramide are a mixture of glucosylceramide with saturated or unsaturated fatty acid residues (C16:0, C18:0, C20:0, C22:0, C23:0, C24:0, C24:1). 3-Phosphoadenosine-5-phosphosulphate (PAPS) was purchased from Bellbrook Labs (No. 2059) in high purity. Other commercial sources of PAPS typically contain significant amounts of PAP and are, therefore, not suitable for the assay^18^. α-Galactosylceramide (KRN7000) was purchased from Avanti Polar Lipids, (Alabaster, AL). β-KRN7000 was synthesised and provided by the laboratory of S. van Calenbergh.

### 2.2. Chemistry

Precoated Macherey-Nagel SIL G/UV254 plates were used for TLC and spots were examined under UV light at 254 nm and further visualised by sulphuric acid-anisaldehyde spray or by spraying with a solution of (NH_4_)_6_Mo_7_O_24_**·**4H_2_O (25 g/L) and (NH_4_)_4_Ce(SO_4_)_4_**·**2H_2_O (10 g/L) in H_2_SO_4_ (10%) followed by charring. Column chromatography was performed on Biosolve silica gel (32–63 µm, 60 Å). NMR spectra were obtained with a Varian Mercury 300 Spectrometer. Chemical shifts are given in ppm (*δ*) relative to the residual solvent signals, in the case of CDCl_3_: *δ* = 7.26 ppm for ^1^H and *δ* = 77.4 ppm for ^13 ^C and in the case of pyridine-d_5_: *δ* = 8.74, 7.58 and 7.22 ppm for ^1^H and *δ* = 149.9, 135.5 and 123.5 ppm for ^13^C. Exact mass measurements were performed on a Waters LCT Premier XE TOF equipped with an electrospray ionisation interface and coupled to a Waters Alliance HPLC system. Samples were infused in a CH_3_CN/HCO_2_H (1000:1) mixture at 10 ml/min.

#### 2.2.1. General procedure for Staudinger reduction and acylation reaction for compounds 24 and 25

To a solution of azide **23** (400 mg, 0.42 mmol, 1 eq.) in 16 ml of tetrahydrofuran (THF) at room temperature, a 1 M solution of PMe_3_ in THF (6.3 ml, 6.3 mmol, 15 eq.) was added dropwise. After stirring for 3 h, 2 ml of H_2_O were added and the reaction mixture was allowed to stir overnight at room temperature. Then the solvent was removed under reduced pressure and the residue was co-evaporated with toluene to afford the crude amine. A mixture of the crude amine, 1-ethyl-3-(3-dimethylaminopropyl)carbodiimide (EDC, 131 mg, 0.84 mmol, 2 eq.) and the appropriate fatty acid (0.63 mmol, 1.5 eq.) in 10 ml of CH_2_Cl_2_ was stirred for 24 h at room temperature. The reaction mixture was diluted with CH_2_Cl_2_, washed with H_2_O (2 × 10 ml) and brine (1 × 10 ml), dried over Na_2_SO_4_, filtered, and evaporated to dryness. Purification by column chromatography using 10% ethyl acetate in hexane gave the desired amides in the indicated yield.

##### 2.2.1.1. [(1*S*,2*S*,3*R*)-1-[[[2,3-Bis-*O*-(phenylmethyl)-4,6-*O*-[(*S*)-phenylmethylene]-α-d-galactopyranosyl]oxy]methyl]-2,3-bis(phenylmethoxy)heptadecyl]-*N*-pentanamide (24)

Yield: 74%. ^1^H NMR (300 MHz, CDCl_3_): *δ* 0.87 (t, *J* = 7.2 Hz, terminal CH_3_), 0.88 (t, *J* = 6.7 Hz, terminal CH_3_), 1.18 − 1.35 (m, 26 H, CH_2_), 1.37–1.53 (m, 2 H, CH_2_), 1.55–1.69 (m, 2 H, CH_2_), 1.85–1.92 (m, 2 H, CH_2_), 3.51–3.57 (m, 1 H, H-4), 3.58 (br s, 1 H, H-4”), 3.74–3.82 (m, 2 H, Hb-1 and H-3), 3.88–3.99 (m, 3 H, Hb-6”, Ha-1 and H-3”), 4.05–4.15 (m, 2 H, H-2” and Ha-6”), 4.18 (d, *J* = 3.3 Hz, 1 H, H-5”), 4.24–4.34 (m, 1 H, H-2), 4.46–4.57 (m, 3 H, CH_2_Ph), 4.60–4.66 (m, 1 H, CH_2_Ph), 4.69–4.80 (m, 3 H, CH_2_Ph), 4.85 (d, *J* = 11.6 Hz, 1 H, CH_2_Ph), 4.96 (d, *J* = 3.5 Hz, 1 H, H-1”), 5.46 (s, 1 H, H-8”), 5.76 (d, *J* = 8.2 Hz, 1 H, NHCO), 7.19–7.42 (m, 23 H, arom H), 7.48–7.55 (m, 2 H, arom H). ^13 ^C NMR (75 MHz, CDCl_3_) *δ* 13.82, 14.11, 22.37, 22.67, 25.82, 27.71, 29.36, 29.68, 29.71, 29.79, 30.27, 31.91, 36.41, 50.35, 62.91, 68.22, 69.41, 71.72, 71.90, 73.28, 73.79, 74.37, 75.69, 76.16, 77.20, 79.44, 79.92, 99.68, 101.00, 126.31, 127.54, 127.57, 127.60, 127.66, 127.71, 127.82, 127.88, 128.08, 128.31, 128.35, 128.43, 128.82, 137.82, 138.40, 138.52, 138.56, 138.66, 172.85.

##### 2.2.1.2. [(1*S*,2*S*,3*R*)-1-[[[2,3-Bis-*O*-(phenylmethyl)-4,6-*O*-[(*S*)-phenylmethylene]-α-d-galactopyranosyl]oxy]methyl]-2,3-bis(phenylmethoxy)heptadecyl]-*N*-non-adecanamide (25)

Yield 84%. ^1^H NMR (300 MHz, CDCl_3_): *δ* 0.86–0.92 (m, 6 H, 2 × terminal CH_3_) 1.16–1.37 (m, 54 H, CH_2_), 1.37–1.55 (m, 2 H, CH_2_), 1.55–1.72 (m, 2 H, CH_2_), 1.81–1.97 (m, 2 H, CH_2_), 3.50–3.57 (m, 1 H, H-4), 3.59 (br s, 1 H, H-4”), 3.74–3.82 (m, 2 H, Hb-1 and H-3), 3.88–3.98 (m, 3 H, Hb-6”, Ha-1 and H-3”), 4.04–4.15 (m, 2 H, H-2” and Ha-6”), 4.18 (d, *J* = 3.1 Hz, 1 H, H-5”), 4.25–4.35 (m, 1 H, H-2), 4.47–4.63 (m, 3 H, CH_2_Ph), 4.65–4.80 (m, 4 H, CH_2_Ph), 4.86 (d, *J* = 11.7 Hz, 1 H, CH_2_Ph), 4.95 (d, *J* = 3.4 Hz, 1 H, H-1”), 5.46 (s, 1 H, H-8”), 5.77 (d, *J* = 8.2 Hz, 1 H, NHCO), 7.22–7.43 (m, 23 H, arom H), 7.48–7.55 (m, 2 H, arom H). ^13^C NMR (75 MHz, CDCl_3_) *δ* 14.11, 22.67, 25.71, 25.83, 29.36, 29.45, 29.71, 29.80, 30.26, 31.91, 36.73, 50.33, 62.93, 68.19, 69.41, 71.72, 71.90, 73.29, 73.81, 74.36, 75.69, 76.15, 77.20, 79.49, 79.85, 99.64, 101.00, 126.31, 127.56, 127.68, 127.82, 127.88, 128.08, 128.29, 128.32, 128.35, 128.43, 128.84, 137.82, 138.40, 138.53, 138.64, 172.89.

#### 2.2.2. General procedure for the debenzylation reaction to prepare 12 and 17

A solution of the protected glycoside (0.06 mmol) in CHCl_3_ (3 ml) and EtOH (9 ml) was hydrogenolysed under atmospheric pressure in the presence of palladium black (35 mg). Upon reaction completion, the mixture was filtered through celite. The filter cake was rinsed with CHCl_3_ and EtOH and the filtrate was evaporated to dryness. After purification by column chromatography (10% →; 18% MeOH in CH_2_Cl_2_), the final compounds were obtained as white powders in the indicated yield.

##### 2.2.2.1. [(1*S*,2*S*,3*R*)-1-[(α-d-Galactopyranosyloxy)methyl]-2,3-dihydroxyheptadecyl]-*N*-pentanamide (12)

Yield: 70%. ^1^H NMR (300 MHz, pyridine-d_5_): *δ* 0.79 (t, *J* = 7.4 Hz, 3H, terminal CH_3_), 0.87 (t, *J* = 6.7 Hz, 3H, terminal CH_3_), 1.16–1.51 (m, 24 H, CH_2_), 1.57–1.79 (m, 3 H, CH_2_), 1.80–1.99 (m, 2 H, CH_2_), 2.21–2.35 (m, 1 H, CH_2_), 2.39 (t, *J* = 7.9 Hz, 2H, CH_2_), 4.27–4.33 (m, 2 H), 4.34–4.46 (m, 4 H), 4.51 (t, *J* = 6.0 Hz, 1 H), 4.56 (d, *J* = 2.7 Hz, 1 H), 4.62–4.71 (m, 2 H), 5.21–5.31 (m, 1 H, H-2), 5.57 (d, *J* = 3.8 Hz, 1 H, H-1”), 6.39 (br s, 6 H, OH), 8.43 (d, *J* = 8.5 Hz, 1 H, NH). ^13 ^C NMR (75 MHz, pyridine-d_5_) δ 14.55, 14.84, 23.25, 23.48, 27.06, 28.93, 30.15, 30.46, 30.54, 30.69, 30.89, 32.67, 34.84, 36.99, 52.06, 63.16, 69.02, 70.85, 71.49, 72.13, 73.03, 73.54, 77.14, 102.01, 173.90. HRMS (ESI) *m*/*z*: calculated for C_29_H_58_NO_9_ [M + H]^+^ 564.4106; found 564.4094.

##### 2.2.2.2. [(1*S*,2*S*,3*R*)-1-[(α-d-Galactopyranosyloxy)methyl]-2,3-dihydroxyheptadecyl]-*N*-non-adecanamide (17)

Yield 42%. ^1^H NMR (300 MHz, pyridine-d_5_): *δ* 0.88 (t, *J* = 6.4 Hz, 6H, 2 × terminal CH_3_), 1.11–1.50 (m, 52 H, CH_2_), 1.60–1.75 (m, 1 H, CH_2_), 1.76–1.99 (m, 4 H, CH_2_), 2.23–2.37 (m, 1 H, CH_2_), 2.46 (t, J = 7.5 Hz, 2 H, CH_2_), 4.30–4.36 (m, 2 H), 4.38–4.48 (m, 4 H), 4.53 (t, *J* = 6.1 Hz, 1 H), 4.56 (d, *J* = 3.1 Hz, 1 H), 4.63–4.72 (m, 2 H), 5.23–5.33 (m, 1 H, H-2), 5.59 (d, *J* = 3.8 Hz, 1 H, H-1”), 6.24 (br s, 6 H, OH), 8.48 (d, *J* = 8.8 Hz, 1 H, NH). ^13 ^C NMR (75 MHz, pyridine-d_5_) *δ* 14.68, 23.34, 26.79, 26.91, 30.02, 30.16, 30.21, 30.27, 30.33, 30.40, 30.42, 30.55, 30.76, 32.53, 34.75, 37.20, 51.85, 63.06, 69.08, 70.71, 71.39, 72.01, 72.89, 73.45, 77.13, 101.94, 173.62. HRMS (ESI) m/z: calculated for C_43_H_86_NO_9_ [M + H]^+^ 760.6297; found 760.6267.

#### 2.2.3. *Synthesis of tert*-butyl-*N*-[(1*S*,2*S*,3*R*)-1-[[[2,3-bis-*O*-(phenylmethyl)-4,6-*O*-[(*S*)-phenylmethylene]-α-d-galactopyranosyl]oxy]methyl]-2,3-bis(phenylmethoxy)heptadecyl]carbamate (26)

To a solution of azide **23** (800 mg, 0.84 mmol) in THF (30 ml) at room temperature, a 1 M solution of PMe_3_ in THF (12.6 ml, 12.6 mmol) was added dropwise. After stirring for 3 h at room temperature, H_2_O (4 ml) was added and the reaction mixture was allowed to stir overnight at room temperature. Then the solvent was removed under reduced pressure and additional co-evaporation with toluene to afford the crude amine. The latter was dissolved in CH_2_Cl_2_ (13 ml) and Et_3_N (3.3 ml) followed by the addition of Boc_2_O (1.1 g, 5.03 mmol). The reaction mixture was stirred overnight at room temperature, evaporated under reduced pressure and purified by column chromatography (0%→20% EtOAc in hexanes) to yield **26** (703 mg, 81%) as a colourless oil. ^1^H NMR (300 MHz, CDCl_3_): *δ* 0.90 (t, *J* = 6.6 Hz, terminal CH_3_), 1.21–1.34 (m, 20 H, CH_2_), 1.43 (br s, 9 H, tBu), 1.47–1.73 (m, 6 H, CH_2_), 3.52–3.58 (m, 1 H, H-4), 3.59 (br s, 1 H, H-5”), 3.72–3.81 (m, 2 H, Hb-1 and H-3), 3.82–3.96 (m, 3 H, Hb-6”, Ha-1 and H-2), 3.96–4.09 (m, 2 H, H-2” and H-3”), 4.09–4.13 (m, 1 H, Ha-6”), 4.15–4.21 (m, 1 H, H-4”), 4.42–4.56 (m, 2 H, CH_2_Ph), 4.56–4.68 (m, 2 H, CH_2_Ph), 4.73–4.88 (m, 5 H, CH_2_Ph and NHCO), 4.95 (d, *J* = 3.1 Hz, 1 H, H-1”), 5.47 (s, 1 H, H-8”), 7.18–7.44 (m, 23 H, arom H), 7.50–7.55 (m, 2 H, arom H). ^13 ^C NMR (75 MHz, CDCl_3_) *δ* 14.11, 22.69, 25.83, 28.01, 28.40, 29.36, 29.65, 29.71, 31.92, 51.68, 62.82, 68.48, 69.41, 71.83, 73.61, 74.51, 75.60, 76.10, 77.20, 79.21, 79.43, 79.75, 99.42, 101.01, 126.31, 127.54, 127.57, 127.79, 127.88, 128.08, 128.26, 128.29, 128.32, 128.35, 128.82, 137.83, 138.47, 138.55, 138.64, 138.73, 155.34. HRMS (ESI) *m*/*z*: calculated for C_64_H_86_NO_10_ [M + H]^+^ 1028.6246; found 1028.6260.

#### 2.2.4. *Synthesis of tert*-butyl-*N*-[(1*S*,2*S*,3*R*)-1-[(α-d-galactopyranosyloxy)methyl]-2,3-dihydroxyheptadecyl]carbamate (27)

A solution of **26** (703 mg, 0.68 mmol) in CHCl_3_ (6 ml) and EtOH (18 ml) was hydrogenolysed under atmospheric pressure in the presence of palladium black (50 mg). Upon reaction completion, the mixture was filtered through celite. The filter cake was rinsed with CHCl_3_ and EtOH and the filtrate was evaporated to dryness. After purification by column chromatography (10% →18% MeOH in DCM), compound **27** (242 mg, 61%) was obtained as a pale yellowish solid. ^1^H NMR (300 MHz, pyridine-d_5_): *δ* 0.88 (t, *J* = 6.4 Hz, 3H, terminal CH_3_), 1.15–1.34 (m, 21 H, CH_2_), 1.37–1.46 (m, 1 H, CH_2_), 1.51 (s, 9 H, tBu), 1.60–1.75 (m, 1 H, CH_2_), 1.80–1.98 (m, 2 H, CH_2_), 2.22–2.35 (m, 1 H, CH_2_), 4.24–4.35 (m, 3 H, Ha-1, H-3”, H-4), 4.38–4.53 (m, 4 H, CH_2_-6”, H-4” and H-3”), 4.57 (d, *J* = 3.0 Hz, 1 H, H-5”), 4.61–4.74 (m, 2 H, H-2” and Hb-1), 4.91–5.01 (m, 1 H, H-2), 5.56 (d, *J* = 3.8 Hz, 1 H, H-1”), 6.41 (br s, 6 H, OH), 7.46 (d, *J* = 9.1 Hz, 1 H, NH). ^13 ^C NMR (75 MHz, pyridine-d_5_) *δ* 14.86, 23.50, 27.03, 29.13, 30.17, 30.48, 30.54, 30.58, 30.68, 30.90, 32.68, 34.87, 53.09, 63.10, 68.94, 70.82, 71.47, 72.16, 72.94, 73.41, 77.24, 79.06, 101.81, 157.14. HRMS (ESI) *m*/*z*: calculated for C_34_H_63_N_2_O_10_ [M + pyridine + H]^+^ 659.4477; found 659.4462.

#### 2.2.5. *Synthesis of* [(1*S*,2*S*,3*R*)-1-[(α-d-galactopyranosyloxy)methyl]-2,3-dihydroxyheptadecyl]-*N*-tetracos-15-enamide (22)

The Boc-protected glycophytosphingosine **27** (150 mg, 0.26 mmol) was dissolved in CH_2_Cl_2_ (20 ml) and 1 M HCl in 90% aqueous AcOH solution (100 μL) was added at room temperature. After 35 min TLC showed incomplete conversion of the starting material and another amount of the HCl solution (100 μL) was added. This process was repeated till TLC (CH_2_Cl_2_/MeOH: 8/2) showed full conversion of the starting material. This required a total addition of 800 μL of the HCl solution. The solvents were removed under reduced pressure and the residue was co-evaporated with MeOH (3 × 5 ml). The resulting crude amine (used without further purification) was dissolved in a biphasic mixture of THF (2.5 ml) and saturated aqueous NaOAc (2.5 ml). In a separate flask, nervonic acid (114 mg, 0.31 mmol) was refluxed for 2 h in oxalyl chloride (4 ml) and the crude formed acyl chloride, obtained after evaporation of the solvent by a stream of nitrogen and subsequent drying on high-vacuum, was dissolved in THF (2.5 ml) and added dropwise to the biphasic mixture. The reaction mixture was stirred for 2 h at room temperature and TLC showed complete conversion of the starting material. Next, the aqueous layer was extracted with THF (3 × 15 ml) and the combined organic layer was dried over Na_2_SO_4_, filtered and evaporated. The crude residue was purified by column chromatography (10% → 16% MeOH in CH_2_Cl_2_) furnishing final compound **22** (79 mg, 37%) as a pale solid. ^1^H NMR (300 MHz, pyridine-d_5_): *δ* 0.86 (dt, *J* = 6.7, 5.4 Hz, 3H, terminal CH_3_), 1.15–1.49 (m, 54 H, CH_2_), 1.55–1.74 (m, 1 H, CH_2_), 1.74–1.98 (m, 4 H, CH_2_), 2.07–2.17 (m, 4 H, CH_2_), 2.21–2.35 (m, 1 H, CH_2_), 2.43 (t, *J* = 7.4 Hz, 2 H, CH_2_), 4.21–4.37 (m, 2 H, H-3, H-4), 4.38–4.48 (m, 4 H, Ha-1, H-3” and CH_2_-6”), 4.53 (t, *J* = 6.3 Hz, 1 H, H-5”), 4.57 (d, *J* = 2.9 Hz, 1 H, H-4”), 4.63–4.73 (m, 2 H, H-2” and Hb-1), 4.96 (br s, 1 H, OH), 5.24–5.33 (m, 1 H, H-2), 5.49–5.55 (m, 2H, CH = CH), 5.59 (d, *J* = 3.7 Hz, 1 H, H-1”), 6.10 (br s, 1 H, OH), 6.21–6.79 (m, 3 H, 3 x OH), 6.96 (br s, 1 H, OH), 8.48 (d, *J* = 8.8 Hz, 1 H, NH). ^13 ^C NMR (75 MHz, pyridine-d_5_) *δ* 14.77, 23.41, 26.89, 26.99, 28.02, 30.03, 30.11, 30.26, 30.31, 30.38, 30.41, 30.49, 30.60, 30.64, 30.86, 32.58, 32.61, 34.81, 37.28, 51.97, 63.14, 69.13, 70.79, 71.48, 72.09, 72.97, 73.52, 77.17, 102.01, 130.73, 173.77. HRMS (ESI) *m*/*z*: calculated for C_48_H_94_NO_9_ [M + H]^+^ 828.6923; found 828.6942.

### 2.3. Biological evaluation

The CST reaction was carried out in a total volume of 50 µl containing 3′-phosphoadenosine-5′-phosphosulphate and galactosylceramide (concentrations of 3′-phosphoadenosine-5′-phosphosulphate and galactosylceramides varied according to assay type) in reaction buffer (10 mM HEPES, 16 mM MgCl_2_, 0.2% (v/v) Triton X-100, pH 7.1). All lipids and Triton X-100 were dissolved in chloroform/methanol (1:1), pipetted into reaction vials, and the chloroform/methanol mixture was removed by drying before adding reaction buffer. Reactions were initiated by the addition of 938 ng of human galactosylceramide sulphotransferase (CST), and then incubated at 37 °C for 30 min. All enzymatic reactions were stopped by heating for 10 min at 60 °C.

Analytical experiments were carried out by using a P/ACE MDQ capillary electrophoresis (CE) system (Beckman Instruments, Fullerton, CA) equipped with a DAD detection system. The capillary temperature was kept constant at 15 °C. The electrophoretic separations were carried out by using fused-silica capillary of 60 cm total length (50 cm effective length) × 75.5 µm (id) × 363.7 µm (od) obtained from Optronis GmbH. The following conditions were applied: *λ*_max_ = 260 nm, voltage = −15 kV, running buffer 75 mM phosphate buffer, 0.002% polybrene, pH 5.6 (adjusted by phosphoric acid), electrokinetic injection (−10 kV, 30 s). The capillary was washed with 0.2 M NaOH for 2 min, and running buffer for 2 min before each injection. Data collection and corrected peak area analysis were performed with the 32 Karat software obtained from Beckman Coulter (Fullerton, CA). Further data analysis was carried out with Graph Pad Prism 4 (Graph Pad Software, Inc. San Diego, CA) and Excel. The human CST enzyme was obtained by heterologous expression in CHO cells in analogy to a described procedure^19^. The CE assay method has previously been reported^18^.

#### 2.3.1. Determination of kinetic parameters for CST

For the determination of kinetic parameters (*K*_m_ and *V*_max_), eight different substrate concentrations were chosen. Negative controls were performed in the presence of heat-inactivated enzyme (10 min, 60 °C). Each analysis was repeated three times in independent experiments.

#### 2.3.2. Investigation of CST inhibitors

For CST inhibitor characterisation, full concentration–inhibition curves were determined by testing a suitable range of inhibitor concentrations, to determine IC_50_ values. *K*_i_ values were calculated according to the Cheng–Prusoff equation^20^. The substrate concentration was 100 µM of galactosylceramide (**3**), and 466 µM of KRN7000 (**18**), respectively, and the concentration of the cofactor PAPS was 30 μM. Substrate conversion was strictly controlled to be below 15%. Negative controls were performed in the presence of heat-inactivated enzyme (10 min, 60 °C). Each analysis was repeated three times in independent experiments.

## 3. Results and discussion

### 3.1. Chemistry

**Scheme 1.**
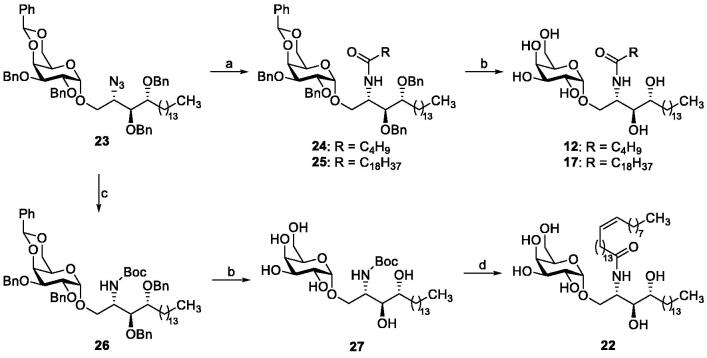
Reagents and conditions: (a) (i) PMe_3_, THF, then H_2_O; (ii) appropriate RCOOH, EDC, CH_2_Cl_2_; (b) H_2_, Pd black; (c) (i) PMe_3_, THF, then H_2_O; (ii) (Boc)_2_O, Et_3_N, CH_2_Cl_2_; (d) (i) HCl, AcOH, H_2_O; (ii) nervonic acid, oxalyl chloride, reflux; the acyl chloride is added to crude amine in THF/aq. NaOAc.

A series of analogues of the natural CST substrate galactocerebroside with variations in the galactose moiety (α- and β-glycosides, substitution of the sugar moiety), in the hydroxylated alkyl chain, and in the fatty acid residue was designed and synthesised. β-KRN7000^21^ and α-glycosides **11**, **13**–**16** and **18**–**21** were prepared as previously described.^22–27^ The synthetic route to obtain α-galactosylceramides **12**, **17** and **22** is depicted in Scheme 1.

The required azidoglycoside **23** was obtained through Lewis acid-catalysed glycosidation reaction as previously reported^28^. Staudinger reduction and subsequent EDC-mediated acylation with the appropriate fatty acid furnished **24** and **25**. Final catalytic hydrogenolysis yielded α-galactosylceramides **12** and **17**. To avoid saturation of the *cis*-double bond of 15-tetracosanoic acid, the amino group generated after Staudinger reduction was Boc-protected prior to the removal of the benzyl groups to afford intermediate **27**. Subsequent removal of the Boc moiety with HCl in acetic acid gave the corresponding psychosine derivative, which was subjected to Schotten–Baumann acylation with 15-tetracosanoyl chloride to afford glycolipid **22**.

### 3.2. Biological evaluation

The synthesised compounds were studied using a previously developed capillary electrophoresis-based assay, in which the conversion of the co-substrate PAPS, acting as a sulphate donor, to adenosine-3′,5′-bisphosphate (PAP) was measured^18^. In addition to the natural substrate galactosylceramide (**3**), which contains a β-galactosyl residue and is present in nerve tissues as a constituent of myelin, the corresponding β-glucosyl derivative glucocerebroside (**9**) was investigated, which is mainly found in liver and spleen. As another naturally occurring sphingolipid psychosine (galactosyl-β-sphingosine, **8**) was investigated, which is a cytotoxic derivative of galactosylceramide (**3**) that lacks its fatty acid residue. Moreover, 13 glycolipids, four of which (**12**, **17**, **20** and **22**) are new compounds, not previously described in literature, were investigated as artificial substrate analogues and/or competitive inhibitors of CST (Table 1). Enzyme kinetic parameters (Michaelis–Menten constant and maximal velocity) were determined for all (artificial) substrates for which significant conversion of >20% as compared to the natural substrate (set at 100%) was observed.

**Table 1.** Investigation of analogues of galactocerebroside as substrates of CST^a^.

Compound	Structure	Percent conversion^a^ at 100 µM	*K*_m_^a^ (µM)	*V*_max_^a^ (µmol/min/mg protein)	*k*_cat_/*K*_m_^a^
**β-Glycosides**					
** 3** Galactosylceramide^b^		100%^c^	**60.3**^18^	**0.0738**^18^	123^18^
** 8** Psychosine		n.d.	**103**^18^	**0.105**^18^	102^18^
** 9** Glucosylceramide^b^		19%	n.d.	n.d.	n.d.
** 10** β-KRN7000		42%	**550**	**0.113**	20.5
**α-Glycosides**					
** 11**		40%	**907**	**0.1080**	11.9
** 12**		30%	**358**	**0.0449**	12.5
** 13**		22%	**392**	**0.0584**	15.0
** 14**		32%	**493**	**0.0590**	12.0
** 15**		20%	n.d.	n.d.	n.d.
** 16**		9%	n.d.	n.d.	n.d.
** 17**		25%	**521**	**0.0519**	10.0
** 18** KRN7000		51%	**438**	**0.0758**	17.3
** 19**		32%	**751**	**0.0834**	11.1
** 20**		15%	n.d.	n.d.	n.d.
** 21**		16%	n.d.	n.d.	n.d.
** 22**		19%	n.d.	n.d.	n.d.

^a^Standard errors were typically below 30% of the reported mean values.

^b^R: saturated or unsaturated alkyl or hydroxyalkyl residue (C16–C27).

^c^Conversion of physiological substrate was set at 100%.

*K*_m_ and *V*_max_ values are shown in bold.

### 3.3. *Structure*–*activity relationships*

#### 3.3.1. Substrates

The natural substrates of CST in nerve tissues are β-galactosylceramides (**3**) which are converted into 3-O-sulphogalactosylcerebroside that constitutes about 4% of total myelin lipids. They are sphingolipids consisting of (i) a sphingosine residue, (ii) a β-galactose, and (iii) a fatty acid residue attached via an amide linkage. Natural galactosylceramide (**3**) may contain different saturated or unsaturated (hydroxy)fatty acid residues with chain lengths mostly between C18 and C27, frequently C24. Galactosylceramide displayed a *K*_m_ value of 60.3 µM determined in our recently developed capillary electrophoresis-based assay and was the best substrate of all compounds investigated in the present study^19^. The corresponding β-glucosylceramide (**9**) were shown to be much weaker substrates with only 19% conversion compared to that of **3** (set at 100%) determined under the same conditions. Another natural sphingolipid that had previously been shown to be a substrate of CST is psychosine (**8**) which is lacking the fatty acid residue of galactosylceramides (**3**). Psychosine was still efficiently sulphurylated by the enzyme showing an only moderately reduced *K*_m_ value of 103 µM in the same assay^19^. A synthetic galactosylceramide analogue, β-KRN7000 (**10**), in which the double bond of the sphingosine core structure is hydrated and, therefore, contains an additional hydroxy group, led to reduced conversion (42% compared to 100% for galactosylceramide) and an increased *K*_m_ value of 550 µM (compared to 60 µM for galactosylceramide) determined under the same conditions. Interestingly, the α-galactoside anomer of compound **10**, KRN7000 (**18**) showed about the same conversion rate and *K*_m_ value indicating that for this series of more polar, hydrated sphingosine-derived synthetic lipids, the enzyme did not discriminate between α- and β-glycosidic configuration. Thus, we investigated further α-galactosyl-lipids (**11**–**17**, **19**–**22**) derived from KRN7000 (**18**) mainly with modification of the fatty acid residue. KRN7000 (**18**) had previously been found to display immunostimulatory and antitumor activity in several in vivo models, and was advanced to clinical trials^29–31^.

**Figure 3.**
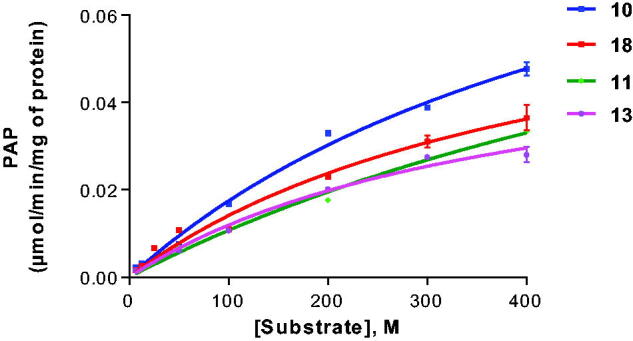
Enzyme kinetics of galactosylceramide sulphotransferase for selected substrates. For *K*_m_ and *V*_max_ values see Table 1.

Using the artificial CST substrate KRN7000 (**18**) as a lead structure, we modified the fatty acid amide moiety. Compound **21**, which lacks the fatty acid residue, was not well accepted as a substrate in this series (19% conversion). We subsequently investigated in a systematic manner the introduction of saturated fatty acid residues with increasing chain length. Compound **11** having a short fatty acid residue, namely acetyl, was tolerated as a substrate (40% conversion) confirming that a long fatty acid residue is not required for interaction with the enzyme and acceptance as a substrate. Probing the optimal length of the fatty acid residue led to the following result: C2 (acetyl, **11**), C5 (**12**), C8 (**13**), C11 (**14**) and C19 (**17**) were about similarly good substrates (Table 1). Medium chain fatty acid residues appeared to be less well tolerated than some shorter chain analogs. In particular, palmitic acid (C16, **16**) showed a sulphurylation rate below 10%. Interestingly, further increase of the chain length in compound **18** (C26) led to the best substrate in this series of α-galactosides with 51% conversion. Aromatic substitution in fatty acid amide analogue **19** was tolerated (compare **19** with **13** and **14**), while the C24-fatty acid containing a *cis*-double bond was not a very good substrate. A bulky aromatic substitution on the sugar moiety in **20** strongly reduced the conversion rate (compare **20** with **18**). Typical Michaelis–Menten curves are shown in Figure 3 for selected substrates (**10**, **11**, **13** and **18**).

#### 3.3.2. Inhibitors

Substrate analogues that were not or only poorly converted by CST with a conversion rate below 20% as compared to galactosylceramide might still be able to bind to the enzyme and thereby act as competitive inhibitors. Therefore, we investigated compounds **9**, **16**, and **10**–**22** for inhibition of CST activity using two different substrates, the natural galactosylceramide (**3**) and the synthetic α-galactoside KRN7000 (**18**). Initial inhibitor screening was performed at 100 µM concentration. Only derivative **16** displayed measurable enzyme inhibition at this concentration (Table 2).

**Table 2.** CST inhibitory activity of galactocerebroside analogues

Compound	Structure	K_i_ ± SEM (µM) versus galactosylceramide (% inhibition at 100 µM)	K_i_ ± SEM (µM) versus KRN7000 (% inhibition at 100 µM)
**β-Glycosides**			
** 9** Glucosylceramide	R = saturated or unsaturated alkyl or hydroxyalkyl residue	(9%)	(−5%)
**α-Glycosides**			
** 16**		**127** ± 12	**159** ± 55
** 20**		(4%)	(1%)
** 21**		(4%)	(−30%)
** 22**		(−7%)	(1%)

**Figure 4.**
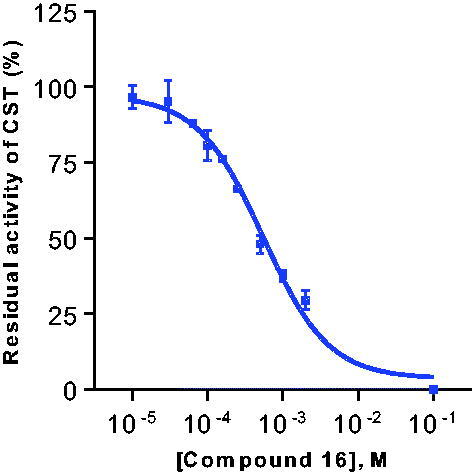
Concentration–inhibition curve of the galactosylceramide sulphotransferase (CST) inhibitor **16**. The curve had to be extrapolated due to limited solubility of **16**. A K_i_ value of 127 ± 12 µM versus galactosylceramide as a substrate was determined.

Subsequently, concentration-dependent inhibition was determined against both substrates, and nearly identical *K*_i_ values were determined against both substrates, 127 µM and 159 µM, respectively (see Table 2 and Figure 4). To our knowledge, this is the first reported substrate-competitive inhibitor of CST.

## 4. Conclusions

In conclusion, we synthesised and investigated selected analogues of the natural CST substrate galactosylceramide (**3**), which were related to the artificial substrate KRN7000 (**18**). Our aim was to study structure–activity relationships for substrates of the enzyme, and to identify competitive inhibitors. We obtained detailed SAR information for the CST galactosylceramide binding site by analysing artificial substrates. Most importantly, a novel, competitive CST inhibitor (**16**) was identified. Compound **16** can serve as a lead structure for optimisation to obtain potent competitive CST inhibitors, which are urgently needed for the treatment of MLD. Our future goal is to develop CST inhibitors for substrate reduction therapy to help MLD patients to survive this devastating genetic disease.
